# 898. Implementation of a Procedure Team in the Burn Intensive Care Unit

**DOI:** 10.1093/jbcr/irag033.394

**Published:** 2026-04-06

**Authors:** Melissa A Loughran, Preema Ponnachan, Timothy Michael Usewicz, Yi T Zhang

**Affiliations:** Temple Burn Center, Temple University Hospital, Philadelphia, PA; Temple University Hospital, Philadelphia, PA; Temple University Hospital, Philadelphia, PA; Temple Burn Center, Temple University Hospital, Philadelphia, PA

## Abstract

**Introduction:**

Facing increased patient acuity, the BICU team performed a needs assessment and formed a Procedure Team, introducing specialized RN and PCA roles. The Procedure RN leads advanced wound care and procedures, while the PCA supports workflow and safety. Together, with the primary RN and provider team, they enhance patient outcomes, staff development, RN retention, and satisfaction, aligning with evidence-based practices and supporting continued improvement in high-acuity care settings.

**Methods:**

Evaluation of efficiency and efficacy over 4 years. The Procedure Team pilot launched in April 2021 to address BICU needs, introducing the Procedure RN role for advanced wound care, multidisciplinary coordination, and staff mentorship. The Procedure PCA role was added to the pilot in 2022 to provide hands-on procedural support and enhance patient safety. Staffing models were adjusted permanently in October 2022 related to the evidence that supported optimal RN-to-patient ratios and resource allocation is evaluated year after year. As patient volume grows, the program continues to expand, onboarding additional RNs and PCAs to support ongoing improvements and workflow efficiency.

**Results:**

The Procedure Team improved procedural efficiency, patient safety, and staff development in the BICU. With specialized RN and PCA roles, workflows became smoother, infection rates dropped to zero for CLABSI and CAUTI, and RN retention increased. Staffing expanded as patient volumes grew, and documentation quality improved. Continuous evaluation and leadership support ensured sustainability and scalability in high-acuity care. The evidence reflects a strong commitment to growth and continuous improvement to recruit and onboard registered nurses, in 2022 we started with17.10 RN FTE’s in burn specialty to 30.9 RN FTE’s in burn specialty in 2025.

**Conclusions:**

The creation and implementation of the Procedure Team in the BICU exemplifies a structured, data-driven, and collaborative approach to nursing innovation. The initiative resulted in measurable improvements in procedural efficiency, infection prevention, documentation quality, and staff competency. Continuous evaluation and expansion, supported by senior leadership, ensured the sustainability and scalability of the program, ultimately enhancing patient care in a high-acuity environment.

**Applicability of Research to Practice:**

The Procedure Team initiative improved procedural efficiency, patient safety, documentation, and staff skills through a structured, data-driven approach. Outcomes align with Evidence-Based Practice and national benchmarks, showing validity and reliability. The scalable model supports ongoing evaluation and adaptation, making it replicable for high-acuity settings and promoting sustainable change in clinical practice.

**Funding for the study:**

N/A.

